# Efficacy and safety of once-weekly tirzepatide for weight management compared to placebo: An updated systematic review and meta-analysis including the latest SURMOUNT-2 trial

**DOI:** 10.1007/s12020-024-03896-z

**Published:** 2024-06-08

**Authors:** Wenhui Qin, Jun Yang, Ying Ni, Chao Deng, Qinjuan Ruan, Jun Ruan, Peng Zhou, Kai Duan

**Affiliations:** 1 Department of Endocrinology and Metabolism, Jingshan Union Hospital of Huazhong University of Science and Technology, Jingshan, China; 2 Department of Pharmacy, Jingshan Union Hospital of Huazhong University of Science and Technology, Jingshan, China; 3 Department of Propaganda, Jingshan Union Hospital of Huazhong University of Science and Technology, Jingshan, China; 4 Department of Vascular Surgery, Wuhan Union Hospital, Huazhong university of science and technology, Wuhan, China; 5 Department of Nephrology, Jingshan Union Hospital of Huazhong University of Science and Technology, Jingshan, China

**Keywords:** Weight loss, Tirzepatide, Placebo, Systematic review, Meta-analysis

## Abstract

**Aim:**

Tirzepatide, a newly developed dual glucose-dependent insulinotropic peptide (GIP) and glucagon-like peptide-1 (GLP-1) receptor agonist, has received approval for treating type 2 diabetes (T2D) and is currently being studied for its potential in long-term weight control. We aim to explore the safety and efficacy of once-weekly subcutaneous tirzepatide for weight loss in T2D or obese patients.

**Methods:**

A comprehensive search was performed on various databases including PubMed, Embase, Cochrane Library, Web of Science, and ClinicalTrials.gov from inception up to April 29, 2024, to identify randomized controlled trials (RCTs) that assessed the efficacy of once-weekly tirzepatide compared to a placebo in adults with or without T2D. The mean difference (MD) and risk ratio (RR) were calculated for continuous and dichotomous outcomes, respectively. The risk of bias was evaluated using the RoB-2 tool (Cochrane), while the statistical analysis was conducted utilizing RevMan 5.4.1 software.

**Results:**

Seven RCTs comprising 4795 individuals ranging from 12 to 72 weeks were identified. Compared to the placebo group, tirzepatide at doses of 5, 10, and 15 mg demonstrated significant dose-dependent weight loss. The mean difference (MD) in the percentage change in body weight (BW) was −8.07% (95% CI −11.01, −5.13; p < 0.00001), −10.79% (95% CI −13.86, −7.71; p < 0.00001), and −11.83% (95% CI −14.52, −9.14; p < 0.00001), respectively. Additionally, the MD in the absolute change in BW was −7.5 kg (95% CI −10.9, −4.1; p < 0.0001), −11.0 kg (95% CI −16.9, −5.2; p = 0.0002), and −11.5 kg (95% CI −16.2, −6.7; p < 0.00001), for the 5, 10, and 15 mg doses, respectively. All three doses of tirzepatide also significantly reduced body mass index and waist circumference. Furthermore, it led to a greater percentage of patients experiencing weight loss exceeding 5, 10, 15, 20, and 25%. Moreover, tirzepatide showed great success in reducing blood pressure, blood sugar levels, and lipid profiles. In terms of safety, gastrointestinal side effects were the most frequently reported adverse events in all three doses of tirzepatide groups, which were generally mild-to-moderate and transient.

**Conclusion:**

Tirzepatide treatment could lead to remarkable and sustained weight loss that is well-tolerated and safe, representing a novel and valuable therapeutic strategy for long-term weight management.

## Introduction

The global obesity pandemic presents a serious and growing public health concern. Projections estimate that by 2035, nearly 2 billion individuals, accounting for 24% of the world’s population, will be classified as obese [1]. China is not immune to this challenge. The rate of obesity in China has risen at a pace more than double the global average since 2010 [2]. By 2018, an estimated 85 million Chinese adults (8.1%) were classified as obese, representing a threefold increase from 2004 [3]. Excess adiposity is associated with a significantly increased risk of numerous complications, including type 2 diabetes(T2D), hypertension, cardiovascular diseases, non-alcoholic steatohepatitis, dyslipidemia, orthopedic issues, and reduced life expectancy [4, 5]. Weight loss of at least 10% has been established as an effective strategy for improving health outcomes and preventing the progression of these obesity-related complications [6].

Due to the limited and poor long-term adherence to weight loss effects of lifestyle interventions and the low public acceptance of bariatric surgery, there has been a longstanding and unmet need for effective anti-obesity medications (AOMs) that can promote body weight management and combat obesity [7, 8]. In recent years, the US Food and Drug Administration (FDA) has approved liraglutide and semaglutide, glucagon-like peptide-1(GLP-1) receptor agonists (GLP-1RAs), for chronic weight management [9]. Notably, glucose-dependent insulinotropic polypeptide (GIP), another enteropancreatic hormone, also contributes to energy balance regulation through cell-surface receptor signaling in the brain and adipose tissue [10]. Clinical studies suggest that co-agonists targeting both GIP and GLP-1 receptors demonstrate a more pronounced effect on glycemia and body weight (BW) compared to selective GLP-1RAs [11].

Tirzepatide (LY3298176), a novel once-weekly subcutaneous dual GIP and GLP-1 receptor agonist, was originally authorized for treating type 2 diabetes. However, it represents a groundbreaking advancement in obesity pharmacotherapy, as it combines gut hormones to achieve substantial weight loss comparable to bariatric surgery [12, 13]. It has now been approved for treatment of T2D in the United States, Europe, Japan, and several other nations and is being investigated for the long-term management of weight in overweight or obese adults. However, it has not been approved for marketing in China. Therefore, we conducted this updated comprehensive review and meta-analysis to systematically explore the efficacy and safety of tirzepatide for weight loss compared to placebo in adults with or without T2D and to provide up-to-date evidence for the clinical promotion of new anti-obesity drugs.

## Methods

### Protocol

This study adhered to the guidelines of Preferred Reporting Items for Systematic Reviews and Meta-Analyses (PRISMA) 2020 for conducting and reporting [14]. Our protocol has been registered in advance in PROSPERO (No. CRD42023458081).

### Search strategy

We comprehensively searched PubMed, Embase, Cochrane Library, Web of Science, and clinical trials registries (https://clinicaltrials.gov) up until April 29, 2024, for randomized controlled trials (RCTs) that compared the efficacy of once-weekly tirzepatide with placebo in adults with T2D or without T2D. No restrictions were imposed based on race, language, or nationality. The following terms were used to conduct the search: (Tirzepatide OR zepbound OR LY3298176 OR Mounjaro OR twincretin OR “dual GIP and GLP-1 receptor agonist”) AND (clinical trials). We adjusted the search strategy according to the requirements of each database.

### Inclusion and Exclusion Criteria

Inclusion criteria were as follows: participants were over 18 years old with or without T2D. The experimental group received once-weekly subcutaneous tirzepatide at specified doses (5,10, and/or 15 mg). The control group received a placebo. Studies with additional control groups were included, but only the placebo-treated control group was analyzed. Studies had to assess weight-related indicators and safety endpoints for an intervention duration of at least 12 weeks. Only RCTs reported in English were considered.

Studies were excluded if they met any of the following criteria: (1) participants did not meet the inclusion criteria; (2) participants were concomitantly using another weight-loss medication; (3) data were incomplete or the full text was unavailable; (4) the study was a duplicate data report, animal experiment, review, comment, conference abstract, trial registry record, meta-analysis, or case report.

### Outcome measures of efficacy and safety

The primary outcome of efficacy was the mean change in BW following the administration of tirzepatide. The additional endpoints were as follows: the proportion of patients who experienced weight loss exceeding 5, 10, 15, 20, and 25% after treatment; the mean changes in body mass index (BMI), waist circumference (WC), fasting plasma glucose (FPG), glycated hemoglobin (HbA1c), fasting lipid profile(total cholesterol [TC], high-density lipoprotein [HDL], low-density lipoprotein [LDL], very low-density lipoprotein [VLDL], triglyceride [TG]), change in systolic blood pressure(SBP) and diastolic blood pressure(DBP). Both primary and secondary outcomes were assessed at the end of treatment (at timepoints ranging from 12 to 72 weeks), and each study’s specific timepoints are listed in Table 1.

**Table 1:** Baseline characteristics of included studies

Study ID	NCT number	Study Groups	Patient (n)	Gender male n (%)	Mean age (year)	BW (kg)	BMI (kg/m^2^)	WC (cm)	HbA1c (%)	duration (weeks)	Journal
Frias [11]	NCT03131687	Tirzepatide 5 mg	55	34 (62%)	57.9 ± 8.2	92.8 ± 19.0	32.9 ± 5.7	110.1 ± 14.83	8.2 ± 1.0	26	Lancet
		Tirzepatide 10 mg	51	30 (59%)	56.5 ± 9.9	92.7 ± 19.5	32.6 ± 5.8	109.6 ± 14.57	8.2 ± 1.1		
		Tirzepatide 15 mg	53	22 (42%)	56.0 ± 7.6	89.1 ± 22.7	32.2 ± 6.2	107.6 ± 15.80	8.1 ± 1.1		
		Placebo	51	29 (57%)	56.6 ± 8.9	91.5 ± 23.1	32.4 ± 6.0	107.7 ± 14.71	8.0 ± 0.9		
Frias [16]	NCT03311724	Tirzepatide 15 mg-1	28	16 (57.1%)	55.5 ± 8.54	88.7 ± 18.21	32.0 ± 5.56	107.0 ± 12.65	8.5 ± 1.17	12	Diabetes, Obesity and Metabolism
		Tirzepatide 15 mg-2	28	23 (82.1%)	56.6 ± 9.21	89.6 ± 16.91	31.1 ± 4.21	105.1 ± 12.19	8.4 ± 1.12		
		Placebo	26	12 (46.2%)	56.0 ± 10.13	89.6 ± 23.70	32.5 ± 5.70	109.1 ± 15.38	8.2 ± 1.22		
Heise [17]	NCT03951753	Tirzepatide 15 mg	45	31 (69%)	61.1 ± 7.1	94.15 ± 13.99	31.28 ± 5.01	NA	7.83 ± 0.72	28	Lancet Diabetes Endocrinol
		Placebo	28	21 (75%)	60.4 ± 7.6	98.74 ± 14.61	32.24 ± 3.96	NA	7.90 ± 0.51		
Rosenstock, (SURPASS-1) [18]	NCT03954834	Tirzepatide 5 mg	121	56 (46%)	54.1 ± 11.9	87.0 ± 21.2	32.2 ± 7.0	NA	7.97 ± 0.84	40	Lancet
		Tirzepatide 10 mg	121	72 (60%)	55.8 ± 10.4	86.2 ± 19.5	32.2 ± 7.6	NA	7.90 ± 0.78		
		Tirzepatide 15 mg	121	63 (52%)	52.9 ± 12.3	85.4 ± 18.5	31.5 ± 5.5	NA	7.85 ± 1.02		
		Placebo	115	56 (49%)	53.6 ± 12.8	84.8 ± 20.0	31.7 ± 6.1	NA	8.05 ± 0.80		
Dahl, (SURPASS-5) [19]	NCT04039503	Tirzepatide 5 mg	116	61 (53%)	62 ± 10	95.8 ± 19.8	33.6 ± 5.9	112.2 ± 15.08	8.3 ± 0.88	40	JAMA
		Tirzepatide 10 mg	119	72 (61%)	60 ± 10	94.5 ± 22.2	33.4 ± 6.2	111.9 ± 15.27	8.36 ± 0.83		
		Tirzepatide 15 mg	120	65 (54%)	61 ± 10	96.3 ± 22.8	33.4 ± 5.9	111.3 ± 15.34	8.23 ± 0.86		
		Placebo	120	66 (55%)	60 ± 10	94.1 ± 21.8	33.2 ± 6.3	109.9 ± 15.34	8.37 ± 0.84		
Jastreboff, (SURMOUNT-1) [12]	NCT04184622	Tirzepatide 5 mg	630	204 (32.4%)	45.6 ± 12.7	102.9 ± 20.71	37.4 ± 6.63	113.2 ± 14.25	5.6 ± 0.36	72	New England journal of medicine
		Tirzepatide 10 mg	636	209 (32.9%)	44.7 ± 12.4	105.8 ± 23.32	38.2 ± 7.01	114.8 ± 15.80	5.6 ± 0.37		
		Tirzepatide 15 mg	630	205 (32.5%)	44.9 ± 12.3	105.6 ± 22.92	38.1 ± 6.69	114.4 ± 15.59	5.6 ± 0.41		
		Placebo	643	207 (32.2%)	44.4 ± 12.5	104.8 ± 21.37	38.2 ± 6.89	114.0 ± 14.92	5.6 ± 0.38		
Garvey, (SURMOUNT-2) [20]	NCT04657003	Tirzepatide 10 mg	312	154 (49%)	54.3 ± 10.7	100.9 ± 20.9	36.0 ± 6.4	114.2 ± 14.1	8.00 ± 0.84	72	Lancet
		Tirzepatide 15 mg	311	152 (49%)	53.6 ± 10.6	99.6 ± 20.1	35.7 ± 6.1	114.6 ± 13.1	8.07 ± 0.99		
		Placebo	315	156 (50%)	54.7 ± 10.5	101.7 ± 22.3	36.6 ± 7.3	116.0 ± 15.7	7.89 ± 0.84		

*BW* body weight, *BMI* body mass index, *WC* waist circumference, *HbA1c* glycated hemoglobin, *n* number of patients, *NA* not applicable

All values are presented as mean ± SD unless otherwise noted

Tirzepatide 15 mg-1: The dose-escalation regimen was 2.5 mg for 2 weeks, followed by 5 mg for 2 weeks, 10 mg for 4 weeks, and then 15 mg for the final 4 weeks

Tirzepatide 15 mg-2: The dose-escalation regimen was 2.5 mg for 4 weeks, followed by 7.5 mg for 4 weeks, and then 15 mg for the final 4 weeks

To assess safety and tolerability, we collected data on the incidence of adverse events (AEs), serious adverse events (SAEs), and discontinuation due to adverse events (DAEs). Additionally, we looked at the proportion of participants experiencing specific events, including nausea, diarrhea, vomiting, constipation, dyspepsia, decreased appetite, and hypoglycemia. Data on the severity of AEs and mortality were also collected.

### Data extraction

Following the specified inclusion and exclusion criteria, three researchers (W.Q., J.Y., and P.Z.) independently screened the literature and extracted data. Disagreements were resolved by a fourth researcher (K.D.). Data extracted included study characteristics (author, publication year, clinical trial registration number, country, study duration, interventions), participants’ baseline characteristics (total sample size, age, gender, race, and anthropometric measures such as BW, BMI, and WC), primary and secondary endpoints of interest, and safety results (e.g., adverse events). For continuous outcomes, we extracted the mean and standard deviation (SD). When SD was unavailable for a specific outcome, we converted it from standard error (SE) or 95% confidence interval (CI). For dichotomous outcomes, we tallied the number of events and the total number in each group. The WebPlotDigitizer4.6 tool (https://automeris.io/documentation.html) was used to extract relevant data that was exclusively displayed in graphical form. Microsoft Excel was used to record all extracted data.

### Quality assessment

The methodological quality of each included RCT was assessed using the Cochrane Collaboration Risk of Bias 2 (RoB-2) tool [15]. The tool assessed five domains: (a) the randomization process, (b) deviations from the intended interventions, (c) missing outcome data, (d) blinding of outcome measurement, and (e) reporting bias. Each study’s risk of bias was categorized as ‘low risk’ (green), ‘some concerns’ (yellow), and ‘high risk’ (red).

The Grading of Recommendations Assessment, Development, and

Evaluation (GRADE) pro-Guideline Development Tool was utilized to assess the certainty of evidence based on the risk of bias, inconsistency in studies, indirectness, imprecision, and publication bias for each tirzepatide dose subgroup. The following outcomes were evaluated: the percentage change (%) and absolute change (kg) in BW from baseline, WC, BMI, the proportion of patients achieving weight loss ≥5, ≥10 or ≥15%, AEs, SAEs, DAEs, nausea, diarrhea, vomiting, and constipation. This method classified the strength of the evidence’s certainty as high, moderate, low, or very low levels.

Three independent authors (W.Q., J.Y., and P.Z.) conducted the quality assessments, with the assistance of a fourth author (K.D.) in the event of any disagreements.

### Data synthesis and analysis

Data were synthesized using Review Manager 5.4.1 software. When combining statistics, the mean difference (MD) was used for continuous variables to represent the effect size, while the risk ratio (RR) was used for dichotomous variables. Confidence intervals (CIs) were estimated using a 95% level. The Higgins I² index was used to assess potential statistical heterogeneity between trials, with a threshold of I² ≥ 50% indicating substantial heterogeneity. Regardless of the I² value, all analyses were conducted using a random-effects model. Forest plots displayed the results of the meta-analysis, while funnel plots were used to assess potential publication bias. We conducted subgroup analyses based on tirzepatide doses (5, 10, and 15 mg) for all endpoints. Additionally, Subgroup analyses based on treatment duration and whether participants had diabetes for the endpoint of changes in BW were also conducted. To assess the robustness of our findings and explore potential sources of heterogeneity, sensitivity analyses were conducted using leave-one-out methods. *P* value of <0.05 was statistically significant.

## Results

### Search results

**Fig. 1:**
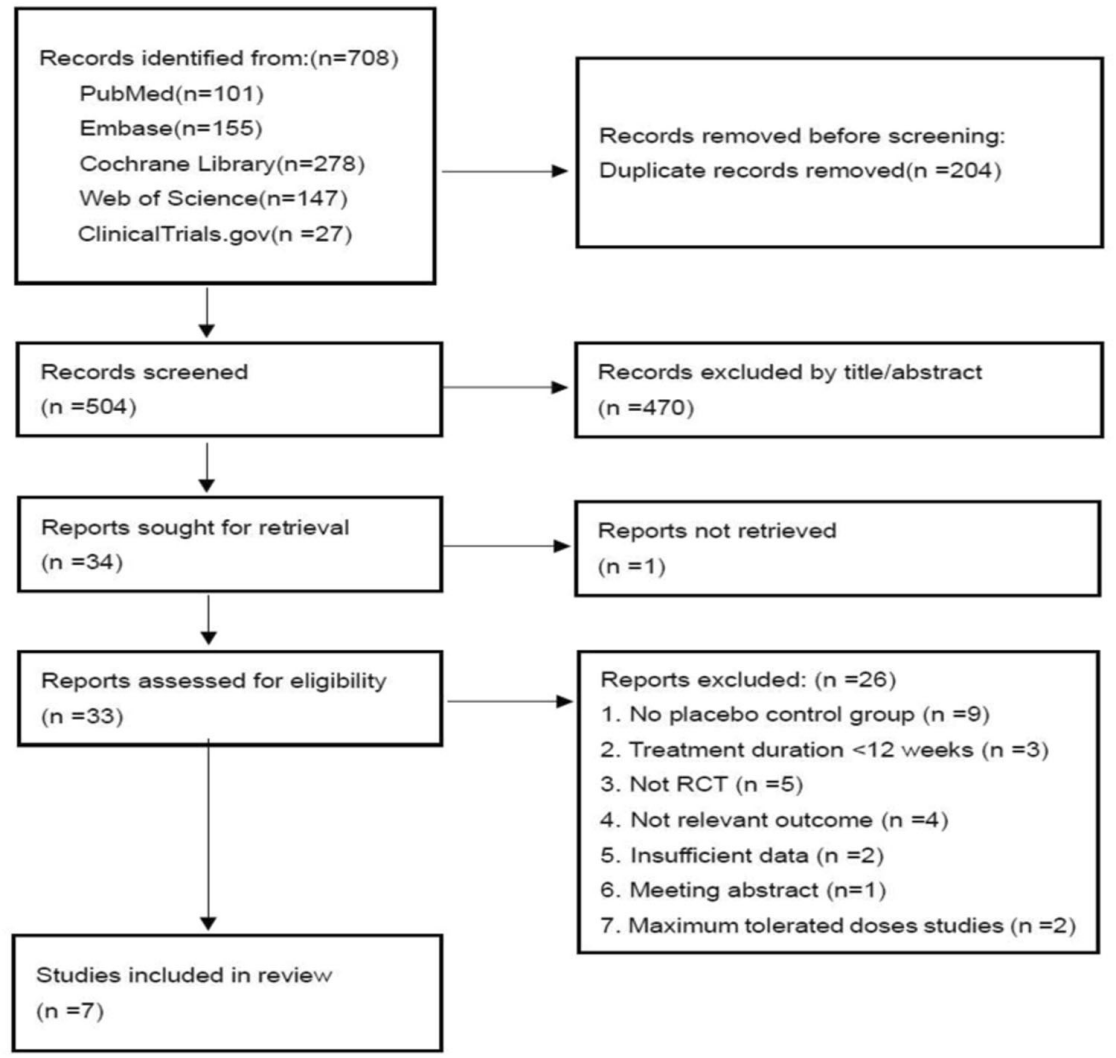
2020 PRISMA flow diagram of the study selection

As shown in Fig. 1, our search strategy identified 708 studies from PubMed (n = 101), Embase (n = 155), Cochrane Library (n = 278), Web of Science (n = 147), and clinicaltrials.gov (n = 27). Following title and abstract screening, 204 duplicate records and 470 studies were identified and removed. A further 27 studies were excluded during the full-text review. Reasons for exclusion included: one study not retrieved (full text unavailable), nine studies lacking a placebo control group, three studies with a treatment duration of less than 12 weeks, five studies that were not RCTs, four studies without relevant outcomes, two studies with insufficient data, one meeting abstract, and two studies focused on maximum tolerated doses. Seven studies [11, 12, 16–20] ultimately met all selection criteria and were included in the meta-analysis.

### Study characteristics

Four of the seven included studies examined the impact of 5 mg tirzepatide, while five studies assessed 10 mg tirzepatide. All seven studies evaluated the effectiveness of 15 mg tirzepatide. In Frias’s trial [16], two groups received the same maintenance dose of 15 mg but with different dose escalation regimens. We combined the outcome data from these two groups into a single treatment group for our analyses. The study included a total of 4795 participants, with 922 (19.2%) receiving a 5 mg dose of tirzepatide, 1239 (25.8%) receiving 10 mg, 1336 (27.9%) receiving 15 mg, and 1298 (27.1%) receiving the placebo. The intervention duration ranged from 12 to 72 weeks across these seven studies. The majority of the research analyzed the effectiveness of tirzepatide in treating T2D, with BW alteration as a secondary outcome, except for SURMOUNT-1 [12] and SURMOUNT-2 [20], which primarily tested tirzepatide for the treatment of obesity. Table 1 provides an overview of the included publications and baseline characteristics of participants.

### Quality assessment

Figure S1 presents the risk of bias evaluation findings for the seven RCTs included in our meta-analysis, as assessed using the RoB-2 tool. Three studies [11, 16, 17] were classified as ‘high risk’ due to missing outcome data. One of these studies [17] was also judged to have a high risk of bias due to deviations from the intended interventions. The evaluation of the remaining four studies [12, 18–20] found them to have low risk in all five domains, indicating an overall low risk of bias for each individual study.

### Main analyses

#### BW changes

**Fig. 2:**
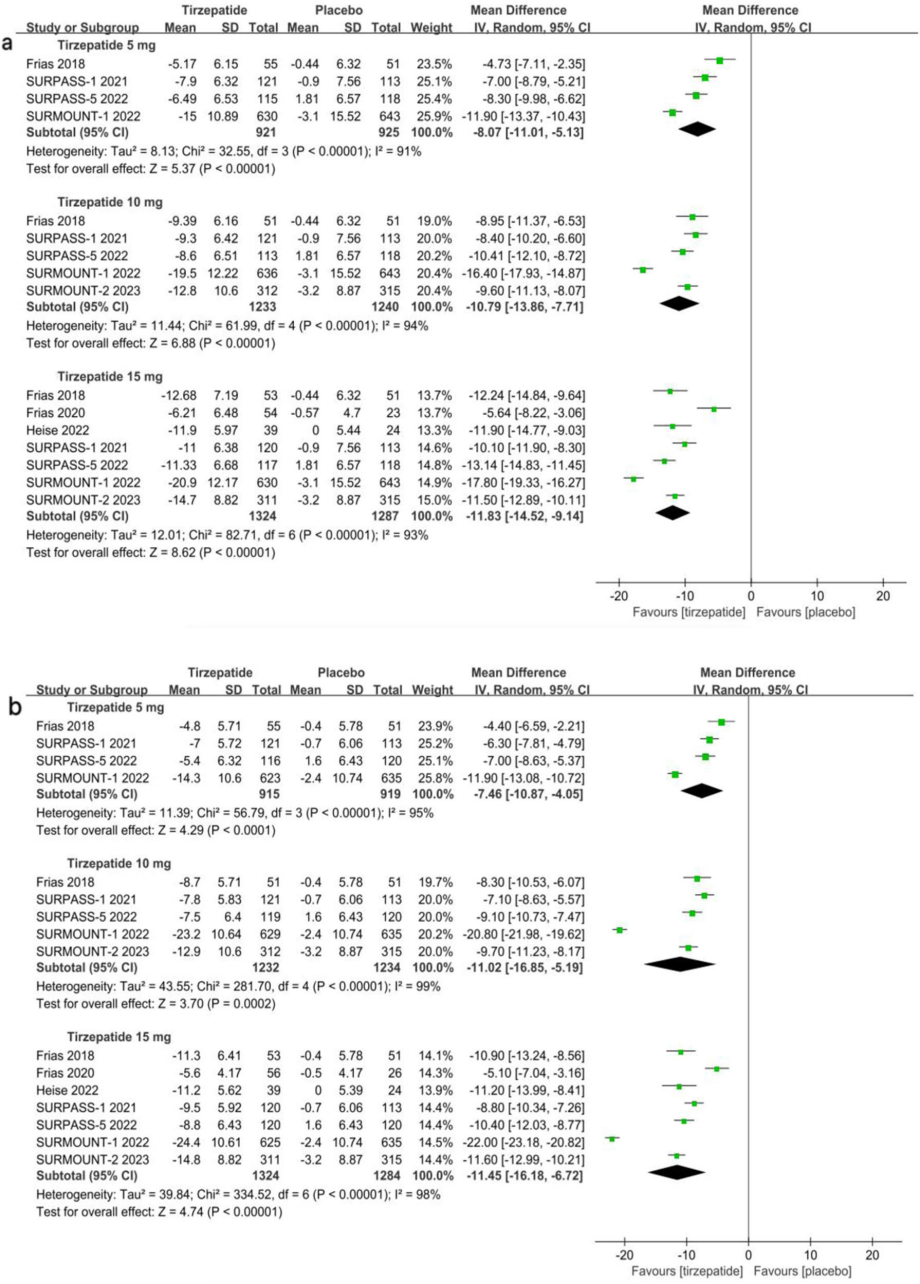
Comparison of three doses of tirzepatide versus placebo for percent weight change from baseline (**a**) and absolute weight change from baseline, in kg (**b**)

The pooled data analysis showed substantial weight reduction in all three tirzepatide doses compared to the placebo group. A dose-dependent relationship was observed for body weight (BW) reduction. Figure 2a and Table S3 demonstrate that the average percentage change in weight exhibited a statistically significant MD compared to the placebo for doses of 5 mg (−8.07, 95%CI [−11.01, −5.13], p < 0.00001, moderate certainty), 10 mg (−10.79, 95%CI [−13.86, −7.71], p < 0.00001, moderate certainty), and 15 mg (−11.83, 95%CI [−14.52, −9.14], p < 0.00001, moderate certainty).

As shown in Fig. 2b and Table S3, the MD in absolute BW change from baseline compared to placebo was −7.5 kg (95%CI [−10.9, −4.1], p < 0.0001, moderate certainty) for 5 mg, −11.0 kg (95%CI [−16.9, −5.2], p = 0.0002, moderate certainty) for 10 mg, and −11.5 kg (95%CI [−16.2, −6.7], p < 0.00001, moderate certainty) for 15 mg of tirzepatide.

#### The proportion of patients achieving weight loss of ≥5, ≥10, ≥15, ≥20 or ≥25%

**Fig. 3:**
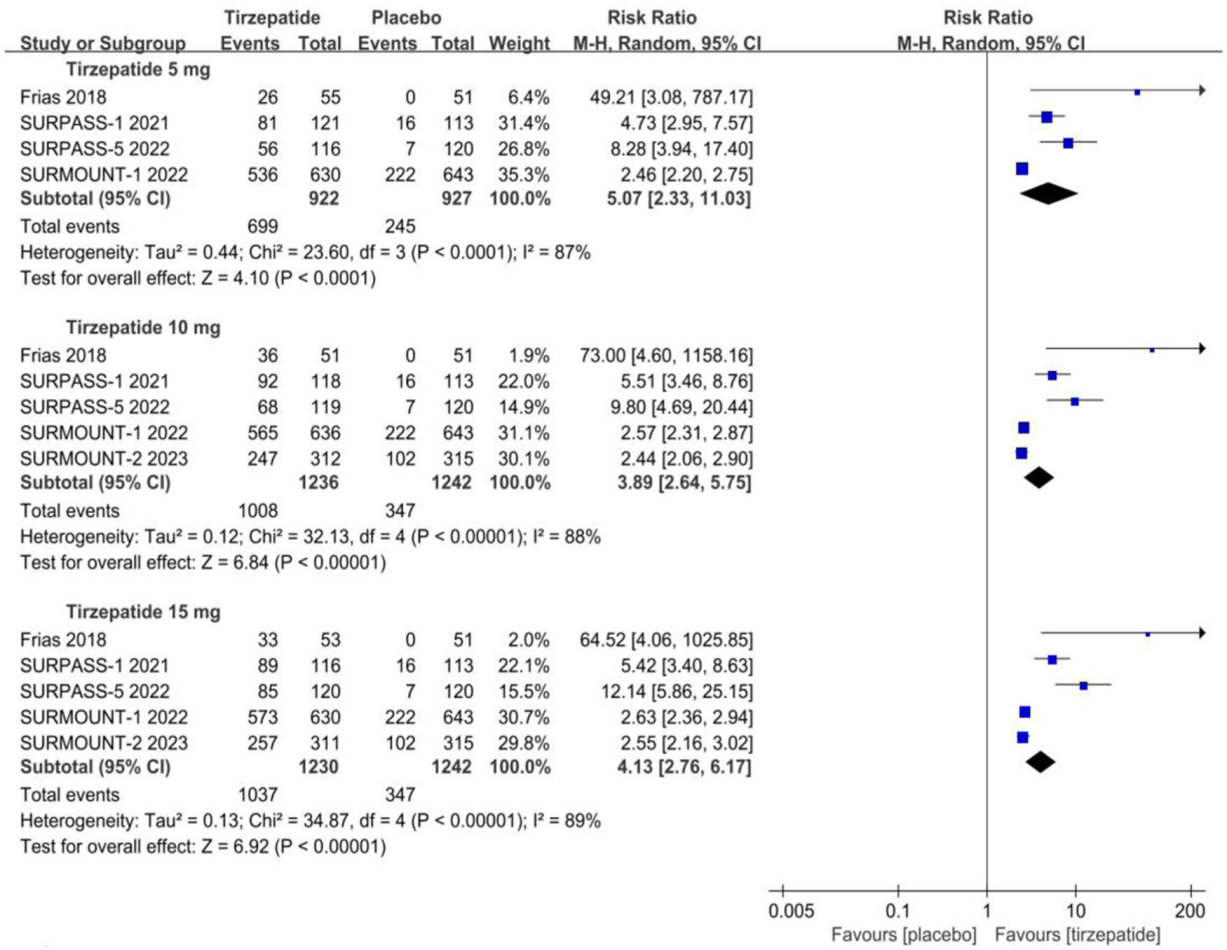
Effect of three doses of tirzepatide on achieving weight loss goals of ≥5% from baseline compared to placebo

Weight loss of ≥5% from baseline is considered a clinically significant response to AOMs [21, 22]. As presented in Fig. 3 and Table S3, a significantly greater proportion of participants in all tirzepatide dose groups achieved at least 5% weight reduction compared to placebo: 75.8% for 5 mg (vs. 26.4%, p < 0.0001, low certainty), 81.6% for 10 mg (vs. 27.9%, p < 0.00001, low certainty), and 84.3% for 15 mg (vs. 27.9%, p < 0.00001, low certainty). This suggests a dose-dependent effect. Similar findings were observed in Figure S2, which showed a higher likelihood of achieving weight loss of ≥10% compared to placebo: 54.4% for 5 mg (vs. 13.3%, p = 0.001, low certainty), 64.9% for 10 mg (vs. 12.3%, p < 0.00001, low certainty), and 69.2% for 15 mg (vs. 12.3%, p < 0.00001, low certainty).

Similar trends were observed for achieving at least 15% weight loss. In Fig. S3 and Table S3, a significantly greater proportion of participants receiving tirzepatide compared to placebo achieved this target: 35.7% for 5 mg (vs. 6.1%, p < 0.00001, moderate certainty), 49.1% for 10 mg (vs. 5.2%, p < 0.00001, moderate certainty), and 54.1% for 15 mg (vs. 5.2%, p < 0.00001, low certainty).

Additionally, two trials SURMOUNT-1 [12] and SURMOUNT-2 [20] which assessed tirzepatide primarily for obesity treatment both reported the proportion of patients achieving weight loss of ≥20 or ≥25%. As shown in Figs. S4–S5 and Table S3, respectively, there were more participants meeting targets compared to placebo for ≥20% weight loss [5 mg (30 vs 3.1%, p < 0.00001, high certainty), 10 mg (40.7 vs 2.4%, p < 0.00001, moderate certainty), 15 mg (48.1 vs 2.4%, p < 0.00001, moderate certainty)] and for ≥25% weight loss [5 mg (15.2 vs 1.6%, p < 0.00001, high certainty), 10 mg (24.6 vs 1.1%, p < 0.00001, moderate certainty), 15 mg (29.3 vs 1.1%, p < 0.00001, moderate certainty)].

#### BMI and WC changes

**Fig. 4:**
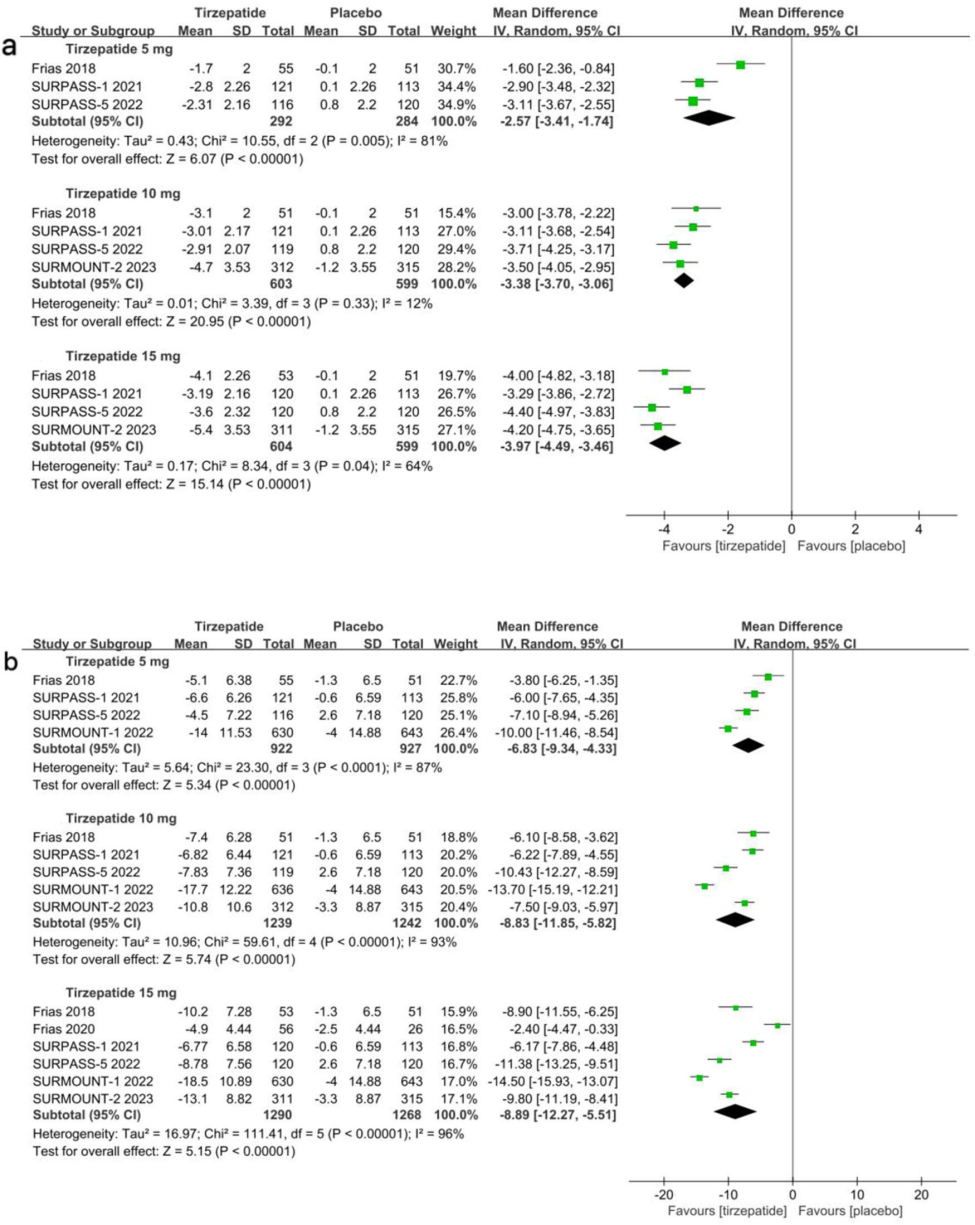
Comparison of three doses of tirzepatide versus placebo for BMI change from baseline, in kg/m^2^ (**a**) and WC change from baseline, in cm (**b**)

Similarly, all tirzepatide doses were superior to placebo in terms of change in BMI from baseline (Fig. 4a and Table S3). A statistically significant MD was observed for the 5 mg dose (−2.6 kg/m^2^, 95%CI [−3.4, −1.7], p < 0.00001, moderate certainty), 10 mg dose (−3.4 kg/m^2^, 95%CI [−3.7, −3.1], p < 0.00001, high certainty), and 15 mg dose (−4.0 kg/m^2^, 95%CI [−4.5, −3.5], p < 0.00001, moderate certainty).

According to the meta-analysis findings shown in Fig. 4b and Table S3, the administration of subcutaneous tirzepatide was also linked to a notable decrease in WC compared to placebo for all three doses, which revealed an MD for 5 mg (−6.8 cm, 95%CI [−9.3, −4.3], p < 0.00001, moderate certainty), 10 mg (−8.8 cm, 95%CI [−11.9, −5.8], p < 0.00001, moderate certainty), and 15 mg (−8.9 cm, 95%CI [−12.3, −5.5], p < 0.00001, moderate certainty).

#### Blood pressure changes

As shown in Figure S6 and Table S3, all tirzepatide doses significantly reduced systolic blood pressure (SBP) compared to placebo. There was a statistically significant MD for the 5 mg dose (−4.86 mmHg, 95%CI [−6.34, −3.37], p<0.00001, moderate certainty), 10 mg dose (−5.3 mmHg, 95%CI [−6.89, −3.72], p<0.00001, low certainty), and 15 mg dose (−6.4 mmHg, 95%CI [−8.13, −4.68], p<0.00001, low certainty).

Compared to the placebo, all three tirzepatide doses resulted in a reduction in DBP. The MD was −1.9 mmHg (95% CI: −4.24, 0.44; P = 0.11, low certainty) for the 5 mg dose, −2.25 mmHg (95% CI: −3.80, −0.69; P = 0.005, low certainty) for the 10 mg dose, and −2.86 mmHg (95% CI: −3.46, −2.27; P < 0.00001, moderate certainty) for the 15 mg dose (Figure S7 and Table S3).

#### HbA1c and glucose changes

All three tirzepatide doses were associated with a significant decrease in HbA1c levels compared to placebo (Figure S8 and Table S3). Mean differences were observed for the 5 mg dose (−1.28%, 95%CI [−2.15, −0.41], p = 0.004, moderate certainty), 10 mg dose (−1.45%, 95%CI [−2.21, −0.69], p = 0.0002, moderate certainty), and 15 mg dose (−1.69%, 95%CI [−2.39, −0.99], p < 0.00001, moderate certainty).

As shown in Figure S9 and Table S3, all tirzepatide doses resulted in a significant reduction in FPG levels compared to placebo. Statistically significant mean differences were observed for the 5 mg dose (−1.89 mmol/L, 95%CI [−3.15, −0.64], p = 0.003, moderate certainty), 10 mg dose (−2.25 mmol/L, 95%CI [−3.36, −1.14], p < 0.0001, moderate certainty), and 15 mg dose (−2.44 mmol/L, 95%CI [−3.41, −1.47], p < 0.00001, moderate certainty).

#### Fasting lipid profile changes

Weekly tirzepatide administration showed a dose-dependent relationship with changes in fasting lipid profiles compared to placebo, as summarized in Table S1 and Table S3. All three doses of tirzepatide significantly decreased TC: −0.25 mmol/L(95% CI :−0.34, −0.15; P <0.00001, high certainty) for 5 mg, −0.30 mmol/L(95% CI:−0.40, −0.20;P <0.00001, high certainty) for 10 mg, and −0.37 mmol/L(95% CI :−0.49, −0.24; P < 0.00001, moderate certainty) for 15 mg. Similar reductions were observed for LDL: −0.15 mmol/L(95% CI :−0.21,−0.09; P <0.00001, high certainty) for 5 mg, −0.19 mmol/L(95% CI:−0.27, −0.10;P <0.0001, high certainty) for 10 mg, and −0.24 mmol/L(95% CI:−0.36, −0.13;P < 0.0001, moderate certainty) for 15 mg. VLDL levels also decreased with all tirzepatide doses: −0.16 mmol/L(95% CI :−0.26, −0.06; P = 0.002, moderate certainty) for 5 mg, −0.19 mmol/L (95% CI: −0.26, −0.12; p < 0.00001, moderate certainty) for 10 mg, and −0.23 mmol/L(95% CI :−0.32, −0.13; P < 0.00001, moderate certainty) for 15 mg. Triglycerides followed the same trend:−0.31 mmol/L(95% CI:−0.46, −0.17; P < 0.0001, low certainty) for 5 mg, −0.37 mmol/L(95% CI:−0.50, −0.25;P < 0.00001, low certainty) for 10 mg, and −0.43 mmol/L(95% CI :−0.54, −0.32; P < 0.00001, low certainty) for 15 mg. The only exception to this trend was HDL, which showed a slight increase for all doses: 0.05 mmol/L (95% CI: −0.00, 0.10; P = 0.07, moderate certainty) for 5 mg,0.06 mmol/L (95% CI :0.02, 0.10; P = 0.008, moderate certainty) for 10 mg, and 0.08 mmol/L (95% CI :0.04, 0.12; P < 0.0001, moderate certainty) for 15 mg.

#### Adverse events

Safety analysis, according to Figure S10, revealed a higher frequency of adverse events (AEs) in the tirzepatide groups compared to placebo. The rates of AEs for tirzepatide were 77.9 vs 69.6% (p < 0.0001, high certainty) for the 5 mg dose, 77.8 vs 71.2% (p = 0.07, moderate certainty) for the 10 mg dose, and 76.4 vs 71.0% (p = 0.03, moderate certainty) for the 15 mg dose (Figure S10 and Table S3). Similarly, a higher proportion of DAEs was observed in the tirzepatide groups compared to placebo, as shown in Figure S12 and Table S3 (5 mg:4.7 vs 2.7%, p = 0.03, moderate certainty;10 mg:6.1 vs 3.0%, p = 0.004, moderate certainty;15 mg:7.3 vs 3.2%, p = 0.002, moderate certainty). Notably, treatment with any of the three tirzepatide doses did not increase the incidence of SAEs compared with placebo, as demonstrated in Figure S11 and Table S3 (5 mg: 6.0 vs 6.4%, p = 0.76, moderate certainty;10 mg: 6.5 vs 6.6%, p = 0.92, moderate certainty; 15 mg: 5.4 vs 6.5%, p = 0.36, moderate certainty). Likewise, there was no significant difference in the occurrence of hypoglycemia between the tirzepatide and placebo groups (Figure S13 and Table S3;5 mg:11.7 vs 10.0%, p = 0.17, low certainty;10 mg: 13.1 vs 10.0%, p = 0.11, low certainty; 15 mg: 12.1 vs 9.5%, p = 0.06, low certainty).

All tirzepatide groups experienced GI AEs, including nausea, diarrhea, vomiting, constipation, dyspepsia, and decreased appetite at a higher frequency compared to placebo (Table S2 and Table S3). RR for tirzepatide compared to placebo were: nausea (5 mg: 2.63, P <0.00001, moderate certainty;10 mg:3.42, P <0.00001, moderate certainty;15 mg: 3.24, P <0.00001, low certainty); diarrhea (5 mg: 2.04, p = 0.007, low certainty;10 mg: 2.29, P <0.00001, moderate certainty;15 mg: 2.38, P <0.00001, low certainty); vomiting(5 mg: 3.95, P <0.00001, moderate certainty; 10 mg: 4.34, P <0.00001, moderate certainty;15 mg: 5.29, P <0.00001, moderate certainty); constipation(5 mg: 3.04, P <0.00001, moderate certainty; 10 mg: 2.83, P <0.00001, moderate certainty; 15 mg: 2.29, P <0.00001, moderate certainty); dyspepsia(5 mg: 2.28, P <0.0001, moderate certainty; 10 mg: 2.44, P <0.00001, moderate certainty; 15 mg: 2.45, P <0.00001, moderate certainty);and decreased appetite(5 mg: 3.21, P <0.00001, moderate certainty; 10 mg: 4.17, P <0.00001, moderate certainty;15 mg: 3.60, P <0.00001, moderate certainty).

An analysis of mortality across all seven included trials (n = 3497 tirzepatide, n = 3471 placebo) found 9 deaths in the tirzepatide group and 6 deaths in the placebo group (Figure S14). Notably, five studies reported no deaths in the tirzepatide arm [11, 16–19]. However, in SURMOUNT-1 [12], the tirzepatide groups experienced some deaths: 4 in the 5 mg group (1 hepatic failure, 2 COVID-19, 1 severe polytrauma), 2 in the 10 mg group (1 homicide, 1 suspected stroke), and 1 in the 15 mg group (COVID-19). Similarly, SURMOUNT-2 [20], reported 2 deaths in the 10 mg tirzepatide group (smoke inhalation, cardio-respiratory arrest). Importantly, investigators did not attribute any of these deaths to tirzepatide treatment.

### Sensitivity analyses

To assess how each study influenced the results, we performed a leave-one-out sensitivity analysis on the percentage change (%) and absolute change (kg) in weight (Table S4-S5). The withdrawal of SURMOUNT-1 had the greatest impact on reducing heterogeneity across all dose subgroups. This is likely because SURMOUNT-1 included only obese patients without comorbid diabetes, unlike the other studies. Research has shown that individuals with both obesity and T2D tend to experience a smaller reduction in weight in response to anti-obesity drugs compared to those without diabetes [23, 24].

### Subgroup analysis

Subgroup analysis based on treatment duration was performed for both percentage (%) and absolute (kg) changes in BW across the three tirzepatide doses compared to placebo (Table S6). This analysis revealed a dose-dependent increase in weight loss with a longer tirzepatide treatment duration. Notably, all three doses of tirzepatide demonstrated significant weight loss compared to placebo regardless of treatment duration. To further investigate the impact of T2D on weight loss, a subgroup analysis was conducted by separating studies enrolling diabetic patients from SURMOUNT-1 (non-diabetic patients). The results showed a greater weight reduction in the SURMOUNT-1 trial (without diabetes) compared to studies that included individuals with diabetes(Table S7).

Data from Table S8 suggests that the incidence of AEs decreased with longer treatment duration, while the occurrence of SAEs did not increase. This finding indicates that most AEs associated with once-weekly subcutaneous tirzepatide are likely to be transient and mild-to-moderate in severity.

### Publication bias

Visual inspection of the funnel plots for the percentage change and absolute change in BW with the 15 mg tirzepatide dose (Figure S15) suggests a potential publication bias in our included trials. This asymmetry might be due to differences in participant inclusion criteria or the limited number of studies included in this meta-analysis (n < 10). Consequently, the Egger test was not employed to formally assess funnel plot asymmetry, as it is not recommended for such small numbers of studies [25].

## Discussion

To our knowledge, this is the first comprehensive review and meta-analysis to include data from the latest SURMOUNT-2 trial, evaluating the efficacy and safety of once-weekly subcutaneous tirzepatide for weight management. Our analysis of seven RCTs involving 4,795 patients revealed that all three tirzepatide doses (5, 10, and 15 mg) were significantly more effective than placebo in reducing BW. Compared to the placebo group, patients receiving tirzepatide achieved substantial weight loss: 8.07% (7.5 kg) with the 5 mg dose, 10.79% (11.0 kg) with the 10 mg dose, and 11.83% (11.5 kg) with the 15 mg dose. Additionally, a significantly greater proportion of participants in all tirzepatide groups achieved the weight loss goal of ≥5% (76% for 5 mg, 82% for 10 mg, and 84% for 15 mg). These findings suggest a dose-dependent effect, with the 15 mg dose demonstrating the most pronounced benefit. In this group, up to 69, 54, 48, and 29% of patients achieved weight reductions of ≥10, ≥15, ≥20, and ≥25%, respectively. The weight loss benefits observed with tirzepatide are noteworthy. Treatment with all doses also led to significant reductions in both BMI and WC. Moreover, tirzepatide demonstrated positive effects on cardiovascular and metabolic risk factors, including improvements in blood pressure, FPG, and fasting lipid profiles. Regarding safety endpoints, GI AEs such as nausea, diarrhea, vomiting, constipation, dyspepsia, and decreased appetite were the most common negative effects that occurred in all tirzepatide groups, which were usually mild-to-moderate and transient. Notably, the administration of tirzepatide did not show any correlation with an increased risk of SAEs or all-cause mortality.

Historically, most older anti-obesity medications approved by the FDA have resulted in modest placebo-adjusted weight loss, ranging from approximately 3 to 8.6%. Furthermore, some of these medications were limited by the severity of their side effects [26]. Semaglutide 2.4 mg, a recently approved selective GLP-1RA for long-term weight management in obese adults, has shown greater effectiveness compared to these earlier AOMs [27]. Tirzepatide is the first novel once-weekly drug that combines the actions of GIP and GLP-1 receptors. It targets tissues not affected by single-agonist agents and integrates activation signals from both GIP and GLP-1 receptor pathways in the brain circuits controlling food intake. This unique mechanism is believed to contribute to weight loss exceeding that achieved with selective GLP-1RAs [28, 29]. Preclinical studies in DIO mice observed greater weight loss, particularly during the initial treatment phase, with tirzepatide at a dose of 10 nmol/kg compared to semaglutide at a dose of 30 nmol/kg. This was associated with a more significant decrease in food consumption, increased fat burning, and indications of slightly higher energy expenditure [28]. Similar findings were observed in two head-to-head clinical trials involving individuals with T2D. These trials demonstrated significantly greater weight loss with tirzepatide compared to selective GLP-1RAs. The estimated treatment differences in weight loss between the 5‐mg, 10‐mg, and 15‐mg tirzepatide groups and the semaglutide 1 mg group were −1.9, −3.6, and −5.5 kg, respectively [30]. Likewise, the mean differences for 5, 10, and 15 mg tirzepatide versus dulaglutide 1.5 mg were –2 ∙ 1, −4∙4, and ‐6 ∙ 2 kg, respectively [11]. These preclinical findings are consistent with the results of multiple RCTs [11, 12, 16–20] that evaluated the impact of tirzepatide on weight loss, and further supported by the results of our meta-analysis.

The ongoing SURMOUNT clinical trial program is particularly noteworthy as it investigates the efficacy of tirzepatide as an anti-obesity medication. Achieving a ≥ 5% reduction in body weight has long been established as a clinically significant benchmark for improving metabolic health [31]. In SURMOUNT-1 [12], a remarkable finding was that the majority of participants (85, 89, and 91%) in the 5, 10, and 15 mg tirzepatide groups, respectively, achieved the ≥5% weight loss benchmark, compared to only 35% in the placebo group. Notably, the 15 mg tirzepatide group in this trial exhibited an average weight decrease of 20.9%, and an impressive 36.2% of participants successfully achieved the≥25% weight-loss goal, approaching the effects observed with bariatric surgery [32, 33]. It is generally understood that people with obesity and T2D have greater difficulty losing significant weight compared to those without T2D [23]. Consistent with this, the degree of weight loss achieved with tirzepatide 10 and 15 mg in individuals with obesity and T2D in SURMOUNT-2(12.8 and 14.7%) was lower than that observed in SURMOUNT-1 without T2D (19·5 and 20·9%, respectively) [12, 20]. Nevertheless, some argue that medications that result in an average reduction of approximately 15% in body weight among obese individuals represent a new generation of AOMs, as this degree of weight loss is considered sufficient to treat or prevent a broader range of obesity-related comorbidities [34]. Long-term weight loss maintenance is the ultimate goal of weight management. Following diet and exercise interventions, patients typically regain nearly 33% of the weight they initially lost within the first year [35]. The SURMOUNT-3 trial demonstrated that for participants who achieved ≥5.0% weight loss after a 12-week intensive lifestyle intervention lead-in period, tirzepatide could contribute an additional significant 18.4% reduction in body weight [36]. Meanwhile, the SURMOUNT-4 trial showed that withdrawing tirzepatide and switching to placebo led to substantial 14.0% regain of lost weight, whereas continued tirzepatide therapy maintained and even slightly augmented the initial 5.5% weight reduction [37]. Based on the findings of the SURMOUNT program, we believe that tirzepatide represents a unique, successful, and effective pharmacological strategy with promising results for individuals suffering from obesity.

In addition to promoting weight loss, our meta-analysis of exploratory secondary outcomes discovered that tirzepatide treatment was accompanied by significantly greater improvements in various cardiometabolic risk factors. These include WC, FPG, blood pressure, and fasting lipid profile. These improvements could translate over time to a decreased risk of metabolic syndrome, cardiovascular disease, hypertension, T2D, non-alcoholic fatty liver disease (NAFLD), and chronic renal disease, ultimately leading to a marked improvement in the health-related quality of life for overweight or obese people [31, 38]. Several trials have been completed or are in progress to assess the impact of tirzepatide on additional obesity-related issues, and the results of these trials are expected to further expand the indications for tirzepatide. As the first research assessing the effects of tirzepatide versus insulin degludec on liver fat content (LFC), visceral adipose tissue (VAT), and abdominal subcutaneous adipose tissue (ASAT) through MRI techniques, the SURPASS-3 MRI trial revealed promising results. All three doses of tirzepatide (5, 10, and 15 mg) resulted in a substantial reduction in LFC (29.78 to 47.11%) compared with insulin degludec (11.17%) after 52 weeks, as well as a statistically greater change in VAT and ASAT volumes [39]. These data provide additional evidence on the metabolic benefits of tirzepatide for NAFLD. Notably, tirzepatide is also being investigated for its cardiovascular safety in patients with T2D at various levels of cardiovascular risk. The ongoing SURPASS-CVOT trial (NCT04255433) is anticipated to yield conclusive answers regarding tirzepatide’s effect on major cardiovascular events when compared to dulaglutide, a long-acting GLP-1 RA that has demonstrated cardioprotective properties in people with T2D who are at high cardiovascular risk [40]. The SUMMIT trial, estimated to be completed in 2024, is designed to evaluate tirzepatide for improvements in all-cause mortality, heart failure events, and a 6- minute walk test in individuals with heart failure [41]. Overall, these ongoing trials suggest that tirzepatide might be the optimal option for obese people suffering from various metabolic diseases.

This meta-analysis also evaluated the safety of weekly tirzepatide based on various AE data. The safety profile observed was consistent with findings from other incretin-based therapies, particularly similar to the safety profile of GLP-1RA like liraglutide and semaglutide used for obesity treatment [27, 42]. GI AEs such as nausea, diarrhea, vomiting, constipation, dyspepsia, and decreased appetite were the most frequently reported events in all three tirzepatide dose groups compared to placebo. This is likely due to the drug’s effect on long-term gastric emptying. However, these GI AEs were mostly mild to moderate in severity, temporary, and tolerable. They typically occurred at treatment initiation or during dose-escalation phases, and gradually diminished over time, rarely leading to treatment discontinuation [12, 18–20]. Therefore, gradual dose escalation in small increments during clinical use of tirzepatide can minimize these side effects and improve tolerability [11, 16, 43]. Notably, previous analyses of the SURPASS trials suggest that tirzepatide-induced sustained and significant weight loss is independent of GI AEs [44], similar to findings with once-weekly semaglutide 2.4 mg [45]. Intriguingly, despite a notable reduction in HbA1c, the incidence of hypoglycemia did not significantly differ between the tirzepatide and placebo groups. This might be explained by the mechanisms through which tirzepatide lowers blood glucose: it stimulates glucose-dependent insulin release from pancreatic β-cells, resulting in a minimal risk of hypoglycemia when used alone [28].Our meta-analysis further found that treatment with tirzepatide did not increase the incidence of SAEs or lead to an increase in all-cause mortality. In summary, tirzepatide demonstrates a promising safety profile, making it a potentially valuable option for pharmacological treatment of obesity.

Tirzepatide’s recent success and clinical application have reignited interest in the search for even more effective and safer AOMs. GLP-1RAs remain a foundational element of all current AOM candidates. Recently, several trials have included multi-targeting agonists of GLP-1R, GIPR, or glucagon receptor (GCGR) for the treatment of both T2D and obesity. There is particular excitement surrounding the potential of combining GLP-1R/GIPR/GCGR agonism as “triple agonists”. A recent phase 2 trial investigating the triple-hormone-receptor agonist Retatrutide (LY3437943), targeting GLP-1R/GIPR/GCGR, yielded impressive results. At the maximum dosage (12 mg) over 48 weeks, this drug produced a remarkable 24.2% reduction in BW in individuals with obesity. Additionally, a staggering 26% of participants in this group achieved a weight loss exceeding 30% of their initial BW [46]. This represents the most significant weight loss effect observed to date. Significant progress is also being made in the development of GLP-1R/GCGR dual agonists like SAR425899. In a nearly month-long therapy, this drug led to a maximum weight reduction of 5.46 kg in individuals with T2D and 5.32 kg in healthy participants [47]. Another promising approach involves combining a long-acting amylin analog with a GLP-1RA. A trial administering multiple doses of cagrilintide alongside semaglutide 2.4 mg for weight management yielded encouraging results. In this trial, overweight participants experienced a significant 17% reduction in BW when given the highest dose (2.4 mg of cagrilintide with semaglutide 2.4 mg) in 20 weeks [48]. The discovery of dual and triple agonists of human gut hormones has opened exciting new avenues for pharmacological weight loss. We eagerly anticipate the future approval of even more effective medications for obesity management.

This study has several limitations that warrant consideration. First, our meta-analysis relied on published, study-level data rather than real-world patient data. Additionally, all RCTs included received support from the pharmaceutical industry. These factors may have led to an overestimation of tirzepatide’s therapeutic benefits due to potential reporting bias. Second, we were unable to quantify or assess the potential influence of the varying exercise and dietary regimens prescribed across the different studies on participant outcomes. Third, with the exception of the SURMOUNT-1 trial, which focused solely on obese patients, six out of the seven included studies involved selected populations with diabetes. This may have resulted in deviations in primary and secondary endpoint data compared to the non-diabetic population. Fourth, due to the novelty of tirzepatide as an AOM, both the sample sizes and the number of included studies were limited. Finally, the majority of the research originated from North and South America, Europe, and Japan, primarily involving participants of Caucasian ethnicity. Thus, to validate the generalizability of these findings, further clinical trials from diverse geographic regions and involving participants of various racial backgrounds are necessary.

## Conclusion

In summary, this updated meta-analysis of seven RCTs involving 4795 participants confirms the significant and sustained, dose-dependent superiority of all three once-weekly tirzepatide doses in promoting weight loss compared to placebo. Furthermore, tirzepatide demonstrates the ability to improve multiple aspects of metabolic syndrome simultaneously, including glycemia, blood pressure, and lipid profiles, while maintaining a well-tolerated safety profile.

## Supplementary information

Supplementary Information

## Data Availability

The research article contains the data that supports the study’s findings. For any more queries, please contact the corresponding author via email or consult the article’s supporting materials.
